# Association of RDW, NLR, and PLR with Atrial Fibrillation in Critical Care Patients: A Retrospective Study Based on Propensity Score Matching

**DOI:** 10.1155/2022/2694499

**Published:** 2022-05-27

**Authors:** Yao-Zong Guan, Rui-Xing Yin, Peng-Fei Zheng, Chun-Xiao Liu, Bi-Liu Wei, Guo-Xiong Deng

**Affiliations:** ^1^Department of Cardiology, Institute of Cardiovascular Diseases, The First Affiliated Hospital, Guangxi Medical University, Nanning, 530021 Guangxi, China; ^2^Guangxi Key Laboratory Base of Precision Medicine in Cardio-Cerebrovascular Disease Control and Prevention, Nanning, 530021 Guangxi, China; ^3^Guangxi Clinical Research Center for Cardio-Cerebrovascular Diseases, Nanning, 530021 Guangxi, China

## Abstract

**Objective:**

Previous studies have shown inconsistent results in relation to the red cell distribution width (RDW), neutrophil to lymphocyte ratio (NLR), and platelet to lymphocyte ratio (PLR) of atrial fibrillation (AF). This retrospective study is aimed at detecting the association of RDW, NLR, and PLR with AF.

**Methods:**

A total of 4717 critical care patients were screened from the Medical Information Mart for Intensive Care- (MIMIC-) III database. The patients were separated into the non-AF and AF groups. The imbalances between the groups were reduced using propensity score matching (PSM). ROC curves were generated to detect the diagnostic value of RDW, NLR, and PLR. Logistic regression analysis was used to detect the risk factors for AF.

**Results:**

A total of 991 non-AF patients paired with 991 AF patients were included after PSM in this study. The RDW level in the AF group was significantly higher than that in the non-AF group (15.09 ± 1.93*vs*. 14.89 ± 1.91, *P* = 0.017). Neither NLR nor PLR showed any significant difference between the two groups (*P* > 0.05 for each). According to ROC curve, RDW showed a very low diagnostic value of AF (AUC = 0.5341), and the best cutoff of RDW was 14.1 (ACU = 0.5257, sensitivity = 0.658, specificity = 0.395). Logistic regression analysis showed that an elevated RDW level increased 1.308-fold (95%CI = 1.077-1.588, *P* = 0.007) risk of AF. Neither elevated NLR nor elevated PLR was a significant risk factor for AF (OR = 0.993, 95%CI = 0.802-1.228, *P* = 0.945 for NLR; OR = 0.945, 95%CI = 0.763-1.170, *P* = 0.603 for PLR).

**Conclusions:**

Elevated RDW level but not NLR or PLR levels is associated with AF. RDW > 14.1 is a risk factor for AF, but its diagnostic capacity for AF is not of great value.

## 1. Introduction

With the improvement of technology and living standards, numerous countries have entered a period of aging. Atrial fibrillation (AF) is a progressive disease, and its prevalence increases with age, leading to cardiac dysfunction and cardiogenic stroke [1, 2]. Early epidemiological research showed that the number of patients diagnosed with AF has reached at 33 billion globally and continues to increase. Moreover, it is estimated that 3-year societal costs were approximately €20,403 to €26,544 for a patient with AF [3, 4]. Although catheter ablation and cryoballoon ablation have achieved great success in the treatment of AF, the mechanism of AF remains unclear [5, 6], and we still need to invest significant methods in the screening, treatment, and management of AF.

Electrocardiography (ECG) is widely used to identify AF, but not all AF occurs during an ECG examination, even during 24-hour or 72-hour monitoring. Peripheral blood is a sample which is easy to obtain, and blood tests can detect latent diseases early. Thus, we have worked for a long time, aiming to identify hematologic markers of AF. Routine blood tests can provide basic information such as red blood cell (RBC), lymphocyte, neutrophil, and platelet count, and based on this information, several parameters like red cell distribution width (RDW), the neutrophil to lymphocyte ratio (NLR), and the platelet to lymphocyte ratio (PLR) can be calculated. RDW is a parameter reflecting the heterogeneity of RBC volume, which is expressed by the coefficient of variation of RBC volume size. The results in a previous follow-up study of mean 13.6 years on 27,124 general individuals suggested that RDW was associated with the incidence of AF. After being adjusted by age and sex, the incidence of AF was significantly associated with RDW (hazard ratio, HR = 1.26; 95% confidence interval, CI = 1.11-1.44 for fourth *vs*. first quartile of RDW), and this association was still strong after adjustment for potential confounding factors, including iron, vitamin B12 and folate intake, cardiovascular disease risk factors, and several hematological parameters (HR = 1.33, 95%CI = 1.16-1.53) [7]. Similarly, another study with 240,477 healthy volunteers followed up to 9 years also showed that higher RDW predicted onset of a wide range of common conditions, including AF. After being adjusted for age, sex, smoking status, educational attainment, hemoglobin, and mean corpuscular volume (MCV), the subgroup with RDW that ranged from 14.5 to 14.9% got a higher risk of AF *vs*. the subgroup with RDW that ranged from 12.5 to 12.9% (HR = 1.91, 95%CI = 1.58-2.30) [8].

NLR is an important indicator of inflammatory activity. A meta-analysis performed by Shao et al. [9] showed that a high NLR was associated with an increased risk of AF recurrence/occurrence with combined odds ratio (OR) of incident AF for baseline NLR level which was 1.25 (95%CI = 1.16-1.35) and 1.518 (95%CI = 1.076-2.142) for the post-NLR level (following coronary artery bypass graft, CABG; radiofrequency catheter ablation, RFCA; and cardioversion). A recent study conducted by Berkovitch et al. [10] revealed that the NLR was directly associated with new-onset AF, and Kaplan-Meier's survival analysis consistently showed that patients with high NLR (≥2.83) had a significantly higher event rate compared with low NLR (<2.83, *P* = 0.006). In contrast, Güngör et al. [11] reported that no significant association was found between elevated NLR and AF (unadjusted OR = 1.21, 95%CI = 0.82-1.76, *P* = 0.32; and OR = 1.33, 95%CI = 0.68-2.58, *P* = 0.40 for gender, hypertension, body mass index (BMI), left atrial volume, hemoglobin, MCV, mean platelet volume, RDW, and high-sensitivity C-reactive protein).

Another blood parameter, PLR, has been associated with new-onset AF after CABG surgery, and PLR levels > 119.3 can predict postoperative AF with 64% sensitivity and 56% specificity (AUC = 0.634, *P* = 0.012) [12]. In addition, another study suggested that after directly current cardioversion, PLR was a risk factor associated with long-term AF recurrence with OR = 3.029 (95%CI = 1.013-9.055, *P* = 0.01) [13]. However, the results from a recent study were different from those of previous studies, suggesting that elevated PLR was not independently associated with AF in patients undergoing isolated CABG (*P* > 0.05, after being adjusted for age, sex, hypertension, and diabetes mellitus) [14].

The associations between NLR, PLR, and AF have been determined previously, but their diagnostic value for AF remains controversial. Although the results of the above studies were adjusted by several covariates, some baseline parameters were imbalanced, which may affect the real results. Patients in critical care always had multiple diseases, one of which was AF. To date, little is known about the association between RDW, NLR, and PLR and AF in critical care patients. Here, this retrospective study is aimed at detecting their relationships based on a large single-center database: Medical Information Mart for Intensive Care-III (MIMIC-III).

## 2. Materials and Methods

### 2.1. Data Source

The MIMIC-III database is a single-center database integrated with information on over 50,000 distinct hospital patients admitted to critical care units during 2001-2012. The demographic characteristics, diagnosis, admission time, death time, laboratory tests, and treatment outcomes were all integrated within 38 tables in this database [15]. Patients' information had been blurred to prevent disclosure of private information. Access to the data of this database requires online training and certification examination online at the National Institutes of Health (NIH). One of the authors (Y.-Z. G.) has completed the training and passed the certification (certification number: 9016236).

### 2.2. Patients Selected and Stratification Method

**Figure 1:**
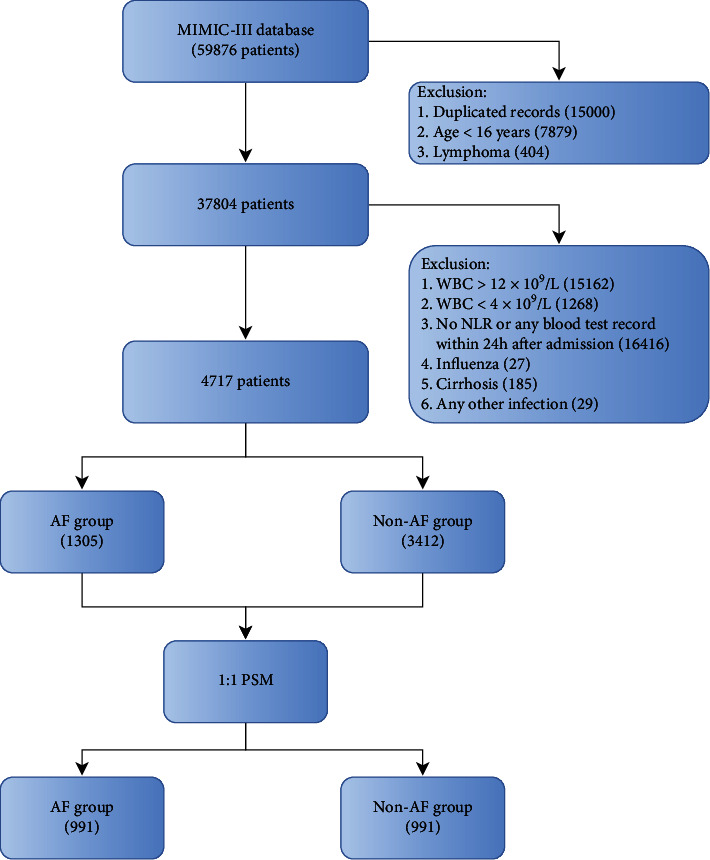
Flowchart of data screening. NLR: neutrophil to lymphocyte ratio; AF: atrial fibrillation; PSM: propensity score matching.

Data from the patients, aged >16 years, who were first admitted to the critical care unit were collected continuously. Routine blood parameters and biochemistry parameters first measured within 24 h after admission were selected. Other records of vasoactive drugs (such as epinephrine, norepinephrine, and dopamine), hemodialysis, and mechanical ventilation used within 24 h after admissions were also selected in this study. Those diagnosed with leukemia and/or lymphoma and/or carried a white blood cell (WBC) count < 4.0 × 10^9^/L or WBC > 12.0 × 10^9^/L were excluded because these factors could affect the accuracy of the result. Patients diagnosed with cirrhosis, influenza, and other infections were also excluded because they would affect neutrophil count, NLR, and platelet count. Patients who had no information on routine blood tests within 24 h after admission were also excluded. The patients finally included were divided into a non-AF group and an AF group, and the diagnosis of AF was defined according to ICD-9. NLR was calculated as neutrophil count/lymphocyte count, and PLR was calculated as platelet count/lymphocyte count. The estimated glomerular filtration rate (eGFR) was calculated based on serum creatinine (sCr) according to a previous study [16]. Structured query language with pgAdmin4 PostgreSQL 9.6 was used to screen out the data (https://www.postgresql.org/). Afterward, a propensity score matching (PSM, 1 : 1 matched) was used to reduce the imbalances of age, sex, and comorbidities between the groups with a caliper width of 0.02, and then, the imbalances of RBC counts, potassium, eGFR, blood urea nitrogen (Bun), smoker, acute myocardial infarction (AMI), and hemodialysis were reduced with a caliper width of 0.01 [17]. The flow chart of this study is shown in Figure 1.

### 2.3. Statistical Analysis

Continuous variables were presented as the mean ± standard deviation (SD) or medians with interquartile range (IQR) (if it was abnormally distributed), and categorical variables were presented as numbers and percentages. Continuous data were compared by one-way *ANOVA* or the *Kruskal*-*Wallis* test (if it was abnormally distributed), and categorical data were compared by the Chi-square test. ROC curves were used to detect the diagnostic value of RDW, NLR, and PLR. The cutoff was determined according to the corresponding value of the largest Youden index (Youden index = sensitivity + specificity − 1). Logistic regression analysis was performed to detect the risk factors for AF. The effect of risk factors was presented as an odds ratio (OR) with a 95% confidence interval (CI). Finally, the Pearson correlation coefficient was used to detect the factors correlated with RDW. The Stata software (version 14.0, USA) was used to perform the analysis, and two-sided *P* < 0.05 was set as significant.

## 3. Results

### 3.1. Patients' Characteristics

Before PSM, a total of 4717 patients were screened from the MIMIC-III database. The AF group included 1305 patients (729 males, 55.9%), and the non-AF group included 3412 patients (1817 males, 53.3%). The distributions of age between the two groups were significantly different (*P* < 0.001). The rates of smoker, obesity, AMI, malignancy, pulmonary embolism (PE), and deep venous thrombosis (DVP) were not significantly different between the non-AF and AF groups (*P* > 0.05). The rates of other combinations were all higher in the AF than in the non-AF groups except for liver diseases (Table 1, *P* < 0.05 for all). After PSM, a total of 991 paired patients were matched between the two groups. The differences in the distribution of age and the rate of combinations between different groups were balanced (Table 2, *P* > 0.05 for all).

### 3.2. Laboratory Parameters and Other Factors

Table 1 shows that the levels of RDW, NLR, PLR, potassium, and Bun; neutrophil count; and the rates of vasoactive drugs use, warfarin, aspirin, and hemodialysis were higher in the AF compared with the non-AF groups (*P* < 0.05 for all). The eGFR level was lower in the AF than that in the non-AF groups (*P* < 0.001, Table 1). After PSM, the differences in neutrophil count; the levels of NLR, PLR, potassium, bun, and eGFR; and the rates of use of vasoactive drugs and hemodialysis were balanced (*P* > 0.05 for all). However, the levels of RDW and the rates of warfarin and aspirin use remained higher in the AF than in the non-AF groups (*P* < 0.001).

### 3.3. ROC Curve of RDW for Diagnosis of AF

**Figure 2:**
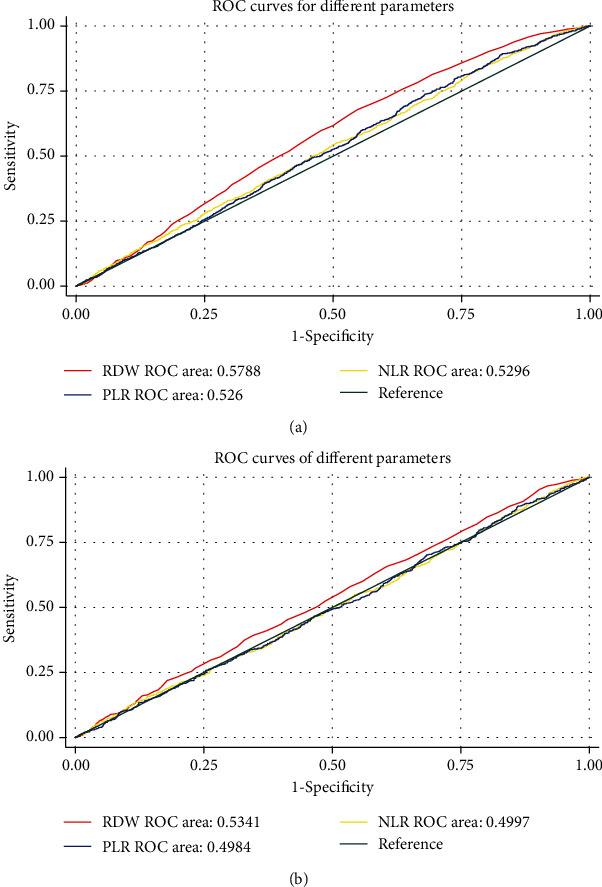
ROC curve of different parameters for atrial fibrillation before and after PSM: (a) before PSM; (b) after PSM. RDW: red cell distribution width; NLR: neutrophil to lymphocyte ratio; PLR: platelet to lymphocyte ratio.

**Figure 3:**
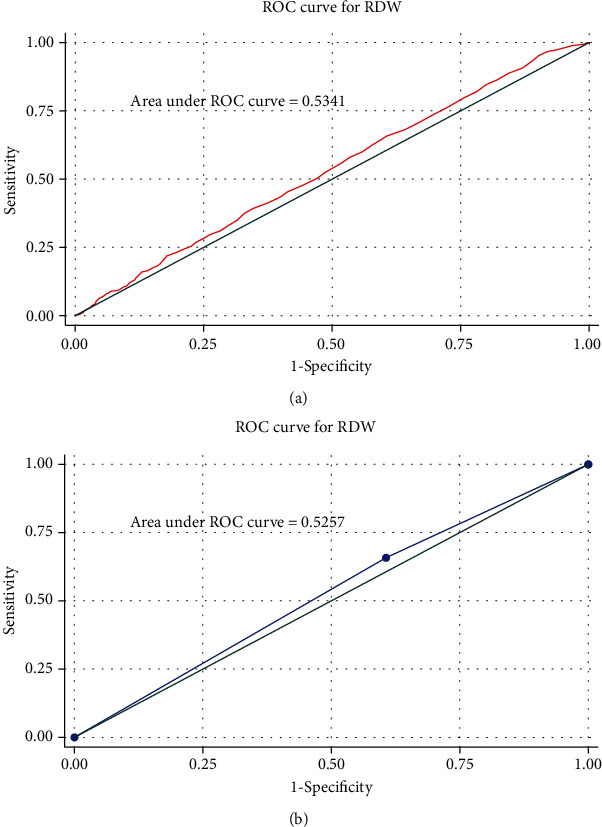
ROC curve of RDW for atrial fibrillation after PSM: (a) ROC curve of RDW after PSM; (b) ROC curve of RDW with the largest Youden index. RDW: red cell distribution width; PSM: propensity score matching.

**Figure 4:**
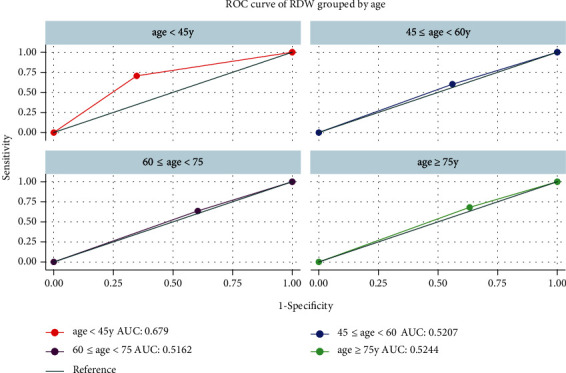
ROC curve of RDW in different age subgroups for atrial fibrillation. AUC: area under ROC curve; RDW: red cell distribution width.

Figures 2 and 3(a) show that before and after PSM, the diagnostic value of elevated RDW levels for AF was low (AUC = 0.5788 before PSM and 0.5341 after PSM). We selected RDW = 14.1 as the cutoff according to the max Youden index and found that the AUC of the ROC curve was 0.5257 (sensitivity = 0.658, 1 − specificity = 0.641, Figure 3(b)). In the subgroup of age distribution, the diagnostic value of RDW for AF was the highest in the age < 45-year group (AUC = 0.6790), but a significant difference was not found among all the subgroups (*P* > 0.05, Figure 4).

### 3.4. Risk Factors for AF

Logistic regression analysis was used to detect the risk factors for AF. RDW was an independent risk factor for AF (OR = 1.248, 95%CI = 1.039-1.499, *P* = 0.018). In addition, the risk decreased after adjusting for age and sex (OR = 1.059, 95%CI = 1.011-1.109, *P* = 0.016), but increased after adjusting for age, sex, combinations, laboratory examination parameters, and use of hemodialysis and mechanical ventilation (OR = 1.308, 95%CI = 1.077-1.588, *P* = 0.007, Table 3).

### 3.5. Related Factors of RDW

**Figure 5:**
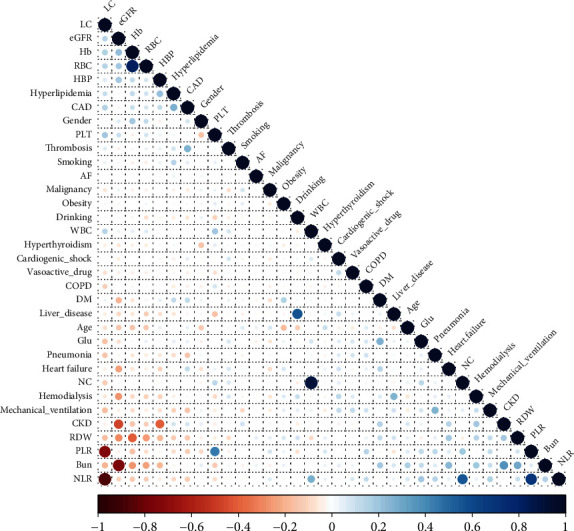
Correlations among the factors. The size of circle presents the value of correlation coefficient; the color of the circle presents the variety of correlation coefficient from -1 to 1. LC: lymphocyte counts; eGFR: estimated glomerular filtration rate; Hb: hemoglobin; RBC: red blood cell; HBP: hypertension; CAD: coronary artery disease; PLT: platelet; AF: atrial fibrillation; DM: diabetes mellitus; Glu: glucose; NC: neutrophil cell counts; CKD: chronic kidney diseases; RDW: red cell distribution width; PLR: platelet to lymphocyte ratio; Bun: blood urea nitrogen; NLR: neutrophil to lymphocyte ratio; COPD: Chronic obstructive pulmonary disease.

According to the results of Spearman analysis, RDW was weakly correlated with AF (*r*^2^ = 0.059, *P* < 0.001). RDW was correlated with several other diseases, such as hypertension (*r*^2^ = −0.188, *P* < 0.001), hyperlipidemia (*r*^2^ = −0.123, *P* < 0.001), and diabetes (*r*^2^ = 0.123, *P* < 0.001), and several laboratory examination parameters, such as lymphocyte count (*r*^2^ = −0.161, *P* < 0.001), platelet count (*r*^2^ = −0.052, *P* = 0.020), and bun level (*r*^2^ = −0.052, *P* < 0.001). The use of mechanical ventilation was correlated with RDW, NLR, and PLR (*r*^2^ = 0.115, 0.168, and 0.114, respectively, *P* < 0.001 for all). The correlations among the factors are shown in Figure 5, and details are provided in the Supplementary Materials.

## 4. Discussion

AF is a high-morbidity cardiovascular disease that leads to deterioration of cardiac function and cardiogenic stroke. Previous studies indicate that AF is highly prevalent in critical care patients and can be served as a marker of poor prognosis and increased mortality [18, 19]. To date, the mechanism of AF has not been well elucidated, but it has been accepted by many experts and scholars that the inflammatory response plays an important role in the formation and maintenance of AF, and decrease of cardiovascular event rates can be found after anti-inflammatory therapy [20, 21]. An increasing number of biomarkers of AF, such as interleukin-6 (IL-6), IL-8, and N-terminal probrain natriuretic peptide (NT-proBNP), have been reported in recent decades [22, 23]. In addition, overload capacity of the atrium results in changes in myocardial electrical and anatomical structure, increasing the risk of AF [24, 25]. In several previous studies, the associations between several hematological parameters, such as RDW, NLR, PLR, and AF, were inconsistent. Our findings indicate that an elevated RDW but neither NLR nor PLR level is significantly associated with AF, and it is an independent risk factor for AF. However, the diagnostic capacity of RDW for AF is not of great value. The data of our study provide more evidence for further research on RDW, NLR, and PLR with AF.

RDW is an easily accessible hematologic parameter, reflecting the distribution of erythrocyte volume. RDW has been indicated as a biomarker of inflammation and is associated with several cardiovascular events, such as myocardial infarction, atherosclerosis, heart failure, AF, and left atrial thrombus [26–31]. Data from a recent study showed that RDW increased with enlargement of the left atrium and was independently associated with AF progression [32]. This result indicated that RDW might reflect the volume of the left atrium. Our findings were consistent to previous reports that the RDW level was significantly higher in the AF group compared with the non-AF groups. This result suggested that the inflammation in the AF group may be more active and reflect the increased RDW levels. What is more, RDW has also been associated with chronic obstructive pulmonary disease (COPD) and stroke [33, 34]. After PSM was balanced, other factors, such as COPD and stroke, were balanced between the AF and the non-AF groups, and the difference in RDW between the two groups remained significant, providing an evidence for the association between RDW and AF. The results of logistic regression analysis show us that RDW may be an independent risk factor for AF, which is similar to those noted in previous reports [35]. In the present study, as shown in Figures 2 and 3, ROC curves did not show a reliable diagnostic value of RDW for AF. This finding may be explained by the fact that RDW can act as a marker of inflammation with low specificity, and the notion can be explored based on its associations with several diseases reported before [36–38]. In addition, RDW was measured as the width (fL) of the erythrocyte distribution curve at a relative height of 20% above the baseline (RDW-SD) in our study but not the coefficient of variation (RDW-CV); the latter was used in many other studies. In contrast to RDW-CV, RDW-SD is independent of MCV, which means the accuracy of the results may be biased because different measures of RDW were used.

It was reported that in general population, the elevated RDW was associated with the all-cause mortality, and the HR increased with the elevation of RDW, even after being adjusted for confounding factors including age, sex, BMI, smoking, diabetes, MCV, and several other parameters. In addition, the results suggested that there were significant differences of BMI and smoker among different RDW subgroups [39]. Elevated RDW was also used as a predictor of poor prognosis of anticoagulation response in patients with AF and the impatient adults with SARS-CoV-2 infection [40, 41]. In a previous study, adjusted OR value of metabolic syndrome was lower at the third and fourth quartile of RDW in males, and RDW might play a mediatory role in the relationship between waist circumference and the dysmetabolic outcomes in obese people [42]. Another large-size study reported that the patients got significantly increased risk of new-onset AF with obesity (HR = 1.327), overweight (HR = 1.123), and upper normal (HR = 1.040) [43]. However, there are also different voices. “Obesity paradox” was used to present that obesity was associated with the reduced risk of all-cause death, and overweight had been associated with significantly reduced risk of AF (HR 0.82, 95%CI = 0.73-0.89, *P* < 0.001) [44]. In the current study, neither before nor after PSM, there was no significant difference in the ratios of obesity between AF and non-AF groups. We also took the effect of obesity on RDW and AF into consideration and included it as the covariate in the analysis of the association of RDW and AF. We found that the RDW in the AF group was still significantly higher than that in the non-AF group. In addition, in the Spearman analysis, the correlation value of obesity and RDW and the value of obesity and AF were small. But, in some degree, however, the number of the obesity was little, and its real effects on the RDW and AF may be underestimated. In addition, the correlation analysis in Figure 5 showed that RDW was correlated with several factors, but their interrelationship needs further determination. Thus, the evidence of using RDW as a sensitive and accurate indicator for the diagnosis of AF is not sufficient. In addition, the exact mechanisms of the increased RDW in AF patients remain to be explored.

NLR and PLR also reveal inflammatory activity, which can be used as a candidate blood-based inflammatory biomarker that is inexpensive and readily available. Bazoukis et al. [22, 23] indicated that the NLR may have the capacity to predict the recurrence of AF in patients undergoing catheter ablation. Recent studies have reported inconsistent findings of the association between a high NLR and postoperative AF. Excessive PLR levels may be a predictor of poor outcomes in patients with cardiovascular disease [45–49]. However, Luo et al. [50] suggested that after mitral valve surgery, recurrent AF patients got a higher NLR and PLR than those nonrecurrence patients, but the diagnostic power of NLR for AF recurrence is not high (AUC = 0.643, 95%CI = 0.513-0.773, *P* = 0.036), and PLR was not a significant predictor of AF recurrence (AUC = 0.620, 95%CI = 0.492-0.748, *P* = 0.079). Similarly, Ding et al. [51] reported that increased NLR was an independent predictor of nonvalvular AF recurrence *vs*. normal NLR after radiofrequency ablation (HR = 1.438, 95% CI: 1.036-1.995, *P* < 0.05). In the present study, the NLR and PLR levels between the AF and the non-AF groups were all different at first analysis. However, after PSM, although the NLR and PLR were highly increased in the AF compared with the non-AF groups, the difference was not significant. Spearman analysis showed that NLR and PLR were correlated with several other factors, such as glucose level, heart failure, and hyperlipidemia, and NLR was also correlated to PLR with a high correlation coefficient = 0.705. In addition, the above parameters were both based on the lymphocyte count, and the Spearman analysis also suggested that lymphocytes were correlated with several diseases and other parameters. Compared to single count of neutrophil, lymphocyte, and thrombocyte, NLR and PLR were more stable, integrating the detrimental effects of neutrophil elevation, thrombocytosis (reflecting inflammation), and lymphopenia (a marker of physiological stress). Thus, although NLR and PLR reflect the activity of inflammation to some degree, these parameters are influenced by many factors. In the present study, the patients were selected from the critical care unit, and these patients always had several other diseases that may affect the inflammatory reaction. Therefore, the diagnostic capability of NLR and PLR for AF and their association with AF may be overestimated or underestimated, and more large-size studies are still needed to expose their relationship.

Both smoking and drinking also were the risk factors for AF. A meta-analysis showed that smoking was associated with AF with a summary relative risk (RR) = 1.14 (95%CI = 1.10-1.20) per 10 cigarettes per day, and RR = 1.16 (95%CI = 1.09-1.25) per 10 pack-years [52]. Another cross-sectional analysis revealed that there was an 18% increase of AF incidence in offspring with every pack/day increase in parental smoking (adjusted HR = 1.18, 95%CI = 1.00-1.39, *P* = 0.04) [53]. In context to alcohol, the HR for one drink (12 g) per day was 1.16 (95%CI = 1.11-1.22, *P* < 0.001), and abstinence from alcohol reduced AF recurrences [54, 55]. The mechanism that how smoking and drinking could increase the risk of AF remains unclear, and one reason to date is that tobacco and alcohol promote atrial myocardial remodeling, increase atrial fibrosis, damage atrial matrix, and then promote the formation of AF [56]. The ratio of smoker was higher in the AF group than in the non-AF group either before or after PSM in our study, though the difference was not significant. When it comes to drinking, the number of drinkers was significantly larger in non-AF than that in AF groups, but the difference was balanced after PSM. Our final analysis suggested that smoking and drinking would not increase the risk of AF. The reason is mainly because we balanced these parameters, aiming to detect the association of RDW, NLR, and PLR with AF without interference from other factors; and the number of drinker was not large enough. In addition, the patients included in the current study were all selected from critical care unit; thus, our results may be inconsistent with those in previous studies.

There are several limitations in the current study. First, the patients selected in this work were obtained from the critical care unit of a single center, and the findings may not apply to other populations. Second, the diagnosis was determined according to ICD-9; some patients may inevitably be left out. Finally, although several imbalance factors were adjusted, some potential factors may still affect these results.

## 5. Conclusions

Increased RDW level (RDW > 14.1) but not NLR or PLR is significantly associated with AF. RDW > 14.1 may serve as an independent risk factor for AF, but its diagnostic capacity for AF is still not large enough.

## Figures and Tables

**Table 1:** Characteristics of the patients before propensity score matching (PSM).

Parameter	Total	Control	AF	*t*/*χ*^2^	*P*
Number	*n* = 4717	*n* = 3412	*n* = 1305		
Gender (male/female)	2546/2171	1817/1595	729/576	0.108	0.110
Age (*n* (%))					
<45 years	691 (14.6)	669 (19.6)	22 (1.7)	644.989	<0.001
45-59 years	1095 (23.2)	965 (28.3)	130 (10.0)		
60-74 years	1378 (29.2)	958 (28.1)	420 (32.2)		
≥75 years	1553 (32.9)	820 (24.0)	733 (56.2)		
Smoker	1514 (32.1)	1088 (31.9)	426 (32.6)	0.248	0.619
Drinker	391 (8.3)	334 (9.8)	57 (4.4)	36.493	<0.001
Complications (*n* (%))					
Hypertension	1928 (40.9)	1354 (39.7)	574 (44.0)	7.226	0.007
Diabetes mellitus	1304 (27.6)	901 (26.4)	403 (30.9)	9.448	0.002
CAD	1266 (26.8)	772 (22.6)	494 (37.9)	111.484	<0.001
Heart failure	1375 (29.1)	704 (20.6)	671 (51.4)	433.154	<0.001
Cardiogenic shock	109 (2.3)	56 (1.6)	53 (4.1)	24.490	<0.001
Hyperlipidemia	1477 (31.3)	984 (28.8)	493 (37.8)	35.065	<0.001
Obesity	119 (2.5)	79 (2.3)	40 (3.1)	2.158	0.142
Hyperthyroidism	414 (8.8)	268 (7.9)	146 (11.2)	13.098	<0.001
Pneumonia	829 (17.6)	563 (16.5)	266 (20.4)	9.823	0.002
COPD	129 (2.7)	82 (2.4)	47 (3.6)	5.095	0.024
Liver disease	351 (7.4)	281 (8.2)	70 (5.4)	11.302	<0.001
Chronic kidney diseases	622 (13.2)	362 (10.6)	260 (19.9)	71.530	<0.001
Malignancy	941 (19.9)	658 (19.3)	28 3(21.7)	3.407	0.065
Thrombosis	1065 (22.6)	708 (20.8)	357 (27.4)	23.566	<0.001
AMI	212 (4.5)	152 (4.5)	60 (4.6)	0.045	0.832
Stroke	175 (3.7)	106 (3.1)	69 (5.3)	12.566	<0.001
PE	154 (3.3)	117 (3.4)	37 (2.8)	1.054	0.305
DVT	96 (2.0)	68 (2.0)	28 (2.1)	0.110	0.740
Other thrombosis	588 (12.5)	385 (11.3)	203 (15.6)	15.787	<0.001
Blood glucose (mg/dL)	138.02 ± 68.17	137.87 ± 68.88	138.52 ± 67.68	-0.713	0.460
RBC count (×10^12^)	3.75 ± 0.71	3.76 ± 0.71	3.73 ± 0.71	0.981	0.327
MCV(fL)	89.90 ± 7.01	89.82 ± 7.15	90.10 ± 6.61	-1.228	0.219
Hemoglobin (g/dL)	11.35 ± 2.09	11.39 ± 2.10	11.25 ± 2.07	2.109	0.035
RDW	14.91 ± 2.10	14.81 ± 2.14	15.19 ± 1.99	-5.658	<0.001
WBC count (×10^9^)	8.40 ± 2.15	8.38 ± 2.16	8.43 ± 2.13	-0.726	0.468
Platelet count (×10^9^)	214.85 ± 98.86	216.23 ± 102.85	211.25 ± 87.49	1.548	0.122
Neutrophil count	6.53 ± 2.16	6.49 ± 2.16	6.63 ± 2.15	-2.083	0.037
Lymphocyte count	1.19 ± 0.70	1.22 ± 0.73	1.13 ± 0.63	4.057	<0.001
NLR	6.01 (6.61)	5.89 (6.50)	6.35 (6.83)	9.938	0.002
PLR	185.54 (186.34)	183.56 (187.69)	190.69 (179.51)	7.646	0.006
Sodium (mmol/L)	138.64 ± 4.84	138.64 ± 4.90	138.62 ± 4.67	0.130	0.896
Potassium (mmol/L)	4.13 ± 0.73	4.09 ± 0.73	4.22 ± 0.71	-5.320	<0.001
eGFR (mL/min/1.73 m^2^)	70.36 (53.00)	76.13(53.87)	57.53 (44.39)	205.801	<0.001
Bun (mg/dL)	19.0 (18.0)	17.0 (16.0)	24.0 (20.0)	241.124	<0.001
SOFA score	4 (4)	3 (4)	4 (4)	54.390	<0.001
Vasoactive drugs (*n* (%))	237 (5.0)	151 (4.4)	86 (6.6)	9.268	0.002
Warfarin (*n* (%))	161 (3.4)	61 (1.8)	100 (7.7)	98.832	<0.001
Aspirin (*n* (%))	700 (14.8)	446 (13.1)	254 (19.5)	30.519	<0.001
Hemodialysis (*n* (%))	240 (5.1)	152 (4.5)	88 (6.7)	10.237	<0.001
Mechanical ventilation (*n* (%))	1454 (30.8)	1037 (30.4)	417 (32.0)	1.079	0.299

CAD: coronary artery disease; AMI: acute myocardial infarction; PE: pulmonary embolism; DVT: deep vein thrombosis; COPD: chronic obstructive pulmonary disease; RDW: red cell distribution width; NLR: neutrophil to lymphocyte ratio; MCV: mean corpuscular volume; PLR: platelet to lymphocyte ratio; eGFR: estimated glomerular filtration rate; Bun: blood urea nitrogen; SOFA: Sequential Organ Failure Assessment.

**Table 2:** Characteristics of the patients after PSM (1 : 1).

Parameter	Total	Control	AF	*F*/*χ*^2^	*P*
Number	*n* = 1982	*n* = 991	*n* = 991		
Gender (male/female)	910/1072	468/532	442/549	1.373	0.241
Age (*n* (%))					
<45 years	40 (2.0)	23 (2.3)	17 (1.7)	5.385	0.146
45-59 years	235 (11.9)	132 (13.3)	103 (10.4)		
60-74 years	671 (33.9)	333 (33.6)	338 (34.1)		
≥75 years	1036 (52.2)	503 (50.8)	533 (53.8)		
Smoker	437 (22.0)	213 (21.5)	224 (22.6)	0.355	0.551
Drinker	94 (4.7)	50 (5.0)	44 (4.4)	0.402	0.526
Complications (*n* (%))					
Hypertension	887 (44.8)	437 (44.1)	450 (45.4)	0.345	0.557
Hyperlipidemia	714 (36.0)	342 (34.5)	372 (37.5)	1.970	0.160
Obesity	55 (2.8)	28 (2.8)	27 (2.7)	0.019	0.891
CAD	736 (37.1)	364 (36.7)	372 (37.5)	0.138	0.710
Heart failure	908 (45.8)	452 (45.6)	456 (46.0)	0.033	0.857
Cardiogenic shock	72 (3.6)	33 (3.3)	39 (3.9)	0.519	0.471
Diabetes mellitus	611 (30.8)	316 (31.9)	295 (29.8)	1.043	0.307
Hyperthyroidism	209 (10.5)	98 (9.9)	111 (11.2)	0.904	0.342
Pneumonia	365 (18.4)	180 (18.2)	185 (18.7)	0.084	0.772
COPD	66 (3.3)	33 (3.3)	33 (3.3)	0.000	1.000
Liver disease	108 (5.4)	58 (5.9)	50 (5.0)	0.627	0.429
Chronic kidney diseases	341 (17.2)	168 (17.0)	173 (17.5)	0.089	0.766
Malignancy	415 (20.9)	196 (19.8)	219 (22.1)	1.612	0.204
Thrombosis	554 (28.0)	276 (27.9)	278 (28.1)	0.010	0.920
AMI	118 (6.0)	61 (6.2)	57 (5.8)	0.144	0.704
Stroke	105 (5.3)	51 (5.1)	54 (5.4)	0.910	0.764
PE	53 (2.7)	24 (2.4)	29 (2.9)	0.485	0.486
DVT	28 (1.4)	11 (1.1)	17 (1.7)	1.304	0.253
Other thrombosis	292 (14.7)	146 (14.7)	146 (14.7)	0.000	1.000
Blood glucose (mg/dL)	140.89 ± 65.08	142.63 ± 65.48	139.15 ± 64.67	1.190	0.234
RBC count (×10^12^)	3.71 ± 0.68	3.72 ± 0.65	3.72 ± 0.72	0.025	0.980
MCV(fL)	90.08 ± 6.84	90.04 ± 7.10	90.12 ± 6.57	-0.236	0.813
Hemoglobin (g/dL)	11.24 ± 2.03	11.24 ± 1.96	11.24 ± 2.10	0.059	0.953
RDW	14.99 ± 1.92	14.89 ± 1.91	15.09 ± 1.93	-2.380	0.017
WBC count (×10^9^)	8.45 ± 2.11	8.49 ± 2.08	8.42 ± 2.13	0.742	0.458
Platelet count (×10^9^)	211.93 ± 94.29	214.21 ± 98.10	209.65 ± 90.31	1.076	0.282
Neutrophil count	6.60 ± 2.14	6.59 ± 2.13	6.61 ± 2.16	-0.227	0.821
Lymphocyte count	1.13 ± 0.66	1.15 ± 0.68	1.12 ± 0.64	0.742	0.458
NLR	6.44 (6.74)	6.45 (6.19)	6.42 (6.54)	0.001	0.980
PLR	194.43(184.31)	195.97(185.57)	190.69(183.71)	0.015	0.903
Sodium	138.80 ± 4.72	138.63 ± 4.64	138.73 ± 4.80	0.643	0.521
Potassium	4.17 ± 0.72	4.17 ± 0.75	4.18 ± 0.67	-0.261	0.794
eGFR	58.61 (44.53)	58.61 (46.69)	58.61 (43.41)	0.182	0.670
Bun	23 (20)	23 (21)	23 (19)	3.037	0.081
SOFA score	4 (4)	4 (4)	4 (4)	2.286	0.131
Vasoactive drugs (*n* (%))	111 (5.60)	59 (5.95)	52 (5.25)	0.468	0.494
Warfarin (*n* (%))	85 (4.3)	16 (1.6)	69 (7.0)	34.528	<0.001
Aspirin (*n* (%))	339 (17.1)	151 (15.2)	188 (19.0)	4.872	0.027
Hemodialysis (*n* (%))	96 (4.8)	51 (5.1)	45 (4.5)	0.394	0.530
Mechanical ventilation (*n* (%))	629 (31.7)	306 (30.9)	323 (32.6)	0.673	0.412

CAD: coronary artery disease; AMI: acute myocardial infarction; PE: pulmonary venous embolism; DVT: deep vein thrombosis; COPD: chronic obstructive pulmonary disease; MCV: mean corpuscular volume; RDW: red cell distribution width; NLR: neutrophil to lymphocyte ratio; PLR: platelet to lymphocyte ratio; eGFR: estimated glomerular filtration rate; Bun: blood urea nitrogen; SOFA: Sequential Organ Failure Assessment.

**Table 3:** Risk factors of atrial fibrillation.

Variables	Unadjusted OR (95% CI)	*P*	Adjusted OR (95% CI)^a^	*P*_a_	Adjusted OR (95% CI)^b^	*P*_b_
Male gender	1.111 (0.931-1.326)	0.241	1.131 (0.947-1.352)	0.175	1.158 (0.964-1.391)	0.118
Age > 75 years	1.129 (0.946-1.347)	0.177	1.147 (0.960-1.371)	0.130	1.114 (0.926-1.340)	0.252
Smoking	1.067 (0.863-1.319)	0.551	1.055 (0.852-1.305)	0.624	1.040 (0.836-1.293)	0.725
Drinking	0.874 (0.577-1.324)	0.526	0.896 (0.590-1.361)	0.606	0.882 (0.576-1.349)	0.562
Hypertension	1.054 (0.883-1.291)	0.477	1.055 (0.884-1.260)	0.551	1.064 (0.883-1.281)	0.517
Diabetes mellitus	0.905 (0.748-1.096)	0.307	0.909 (0.751-1.101)	0.331	0.856 (0.701-1.047)	0.130
Coronary artery diseases	1.035 (0.863-1.242)	0.710	1.022 (0.851-1.227)	0.816	1.019 (0.830-1.250)	0.860
Hyperlipidemia	1.140 (0.949-1.370)	0.295	1.140 (0.949-1.370)	0.162	1.162 (0.952-1.418)	0.139
Obesity	0.964 (0.564-1.647)	0.891	1.042 (0.605-1.795)	0.883	1.052 (0.602-1.838)	0.860
Heart failure	1.016 (0.852-1.213)	0.857	1.027 (0.860-1.226)	0.772	0.999 (0.829-1.205)	0.994
Cardiogenic shock	1.189 (0.742-1.907)	0.472	1.210 (0.754-1.941)	0.430	1.2360 (0.759-2.012	0.394
Pneumonia	1.034 (0.824-1.298)	0.754	1.034 (0.824-1.299)	0.771	1.015 (0.798-1.291)	0.903
COPD	1.000 (0.612-1.634)	1.000	0.995 (0.608-1.627)	0.983	1.025 (0.621-1.693)	0.923
Hyperthyroidism	1.149 (0.862-1.532)	0.342	1.164 (0.869-1.558)	0.308	1.156 (0.861-1.552)	0.336
Thrombosis	1.010 (0.830-1.229)	0.920	1.009 (0.829-1.228)	0.930	1.011 (0.822-1.243)	0.918
Hemodialysis	0.877 (0.581-1.322)	0.530	0.889 (0.588-1.342)	0.575	0.847 (0.553-1.298)	0.446
Mechanical ventilation	1.082 (0.896-1.308)	0.412	1.088 (0.900-1.315)	0.384	1.099 (0.896-1.349)	0.366
RDW > 14.1	1.248 (1.039-1.499)	0.018	1.059 (1.011-1.109)	0.016	1.308 (1.077-1.588)	0.007
NLR ≥ 6.44	0.984 (0.825-1.173)	0.857	1.001 (0.993-1.011)	0.772	0.993 (0.802-1.228)	0.945
PLR ≥ 194.43	0.964 (0.809-1.150)	0.686	1.000 (0.999-1.000)	0.905	0.945 (0.763-1.170)	0.603

COPD: chronic obstructive pulmonary disease; RDW: red cell distribution width; NLR: neutrophil to lymphocyte ratio; PLR: platelet to lymphocyte ratio. *P*_a_: adjusted by age and gender; *P*_b_: adjusted by age, gender, smoking, drinking, combinations, laboratory examination parameters, use of hemodialysis, and mechanical ventilation.

## Data Availability

Full data set is available from the author at guan_yz1007@163.com. However, reanalysis of the full data needs to be approved by MIMIC-III Institute.

## Abbreviations

AF: Atrial fibrillation
RDW: Red cell distribution width
NLR: Neutrophil to lymphocyte ratio
PLR: Platelet to lymphocyte ratio
MIMIC: Medical Information Mart for Intensive Care
PSM: Propensity score matching
AMI: Acute myocardial infarction
PE: Pulmonary embolism
DVT: Deep vein thrombosis
COPD: Chronic obstructive pulmonary disease
CKD: Chronic kidney disease
SOFA: Sequential Organ Failure Assessment
Bun: Blood urea nitrogen
sCr: Serum creatinine
eGFR: Estimated glomerular filtration rate
SD: Standard deviation
IQR: Interquartile range
CVD: Cardiovascular diseases
OR: Odds ratio
HR: Hazard ratio
RR: Relative risk
CI: Confidence interval
AUC: Area under the curve.

## Supplementary Materials

Supplementary Materials Correlations among the factors.txt.
